# Withania somnifera as an Adjunctive Treatment for Refractory Restless Legs Syndrome in Parkinson’s Disease: A Case Report

**DOI:** 10.7759/cureus.20775

**Published:** 2021-12-28

**Authors:** Kaustubh S Chaudhari, Rakesh R Tiwari, Saurabh S Chaudhari, Swati V Joshi, Harish B Singh

**Affiliations:** 1 Department of Internal Medicine, Dr. V.M. Government Medical College, Solapur, IND; 2 Department of Samhita Siddhanta, Smt. K.G. Mittal Punarvasu Ayurvedic College, Mumbai, IND; 3 Department of Internal Medicine, Seth G.S. Medical College, Mumbai, IND; 4 Department of Kayachikitsa, Smt. K.G. Mittal Punarvasu Ayurvedic College, Mumbai, IND

**Keywords:** withania somnifera, insomnia, sleep disorders, restless legs syndrome, parkinson’s disease

## Abstract

Non-motor symptoms of Parkinson’s disease (PD), such as insomnia and restless legs syndrome (RLS), tend to worsen and become refractory as neurodegeneration progresses. We report the case of a 72-year-old female with a six-year history of PD and two-and-half-year history of insomnia and refractory RLS. We added a neuroprotective agent, *Withania somnifera*, to the existing treatment regimen for her insomnia. Besides the partial remission of her insomnia and motor symptoms of PD, there was a complete reversal of the RLS symptoms. *Withania somnifera* has been shown to improve PD symptoms by preventing oxidative damage of the nigrostriatal dopaminergic neurons and improving dopamine levels in the midbrain and corpus striatum. Our case provides the first-time evidence where *Withania somnifera* added for insomnia caused a complete remission of refractory RLS, possibly due to its anti-apoptotic and pro-dopaminergic actions. *Withania somnifera* could prove beneficial in cases where the disease advances but further addition of dopamine agonists for refractory RLS is not possible due to the risk of dopamine augmentation.

## Introduction

The non-motor symptoms of Parkinson’s disease (PD) impair the patients' quality of life far more than its motor symptoms. Sleep disturbances and depression are seen in 60% and 14-35% of PD cases respectively [1]. Sleep disturbances like insomnia and restless legs syndrome (RLS) occur in two-thirds of community-dwelling PD cases [2]. Conventional treatment targets neurotransmitters involved in PD. Hence, with progressing pathology, the symptoms become refractory to these medications. This necessitates the addition of molecules that could stall neurodegeneration. *Withania somnifera*, a plant-derived neuroprotective and pro-dopaminergic agent, has been shown to improve sleep in human and animal studies [3,4]. In this report, we present the case of an elderly female with PD with insomnia and refractory RLS whose symptoms were relieved possibly due to the addition of *Withania somnifera* to the existing therapy.

## Case presentation

A 72-year-old woman diagnosed with PD six years back experienced resting tremors in both hands that responded well to the levodopa-carbidopa combination. However, with time, tremors worsened in amplitude, and she developed rigidity, bradykinesia, inability to write, and frozen gait, which incapacitated her from performing her daily activities. About two-and-half years ago, she reported increased sleep latency, sleep fragmentation (three to four waking episodes/night), early awakening, with effective nighttime sleep reduced to two to three hours, excessive daytime sleepiness, and compromised daytime function. She also developed cramps, tingling, and uneasiness in both legs while sitting during late evenings. This was accompanied by an irresistible urge to move the legs. Moving her legs partially relieved her of these RLS symptoms. She received cognitive behavioral therapy, zopiclone, and clonazepam for insomnia. She had already been receiving pregabalin for her RLS before approaching our clinic. MRI of the brain done at the time showed age-related small vessel changes that did not correlate with the patient’s symptoms. She had mild microcytic hypochromic anemia (hemoglobin: 9.2 gm/dL, serum ferritin: 22 µg/L, mean corpuscular hemoglobin: 23 pg, mean corpuscular volume: 72 fL), which was corrected by giving ferrous sulfate. While her anemia was corrected completely, her sleep disturbances were not fully corrected and recurred about a year ago.

She approached our outpatient service two months back. We ruled out differentials for RLS (periodic limb movement disorder, akathisia, positional discomfort, and nocturnal leg cramps), and insomnia (obstructive sleep apnea, circadian rhythm sleep disorders, pain syndromes, acid reflux, and depression). *Withania somnifera* in the form of root powder linctus (200 mg twice daily after meals) was added to her existing treatment regimen for insomnia. After two months of treatment, the patient reported complete cessation of RLS symptoms, from thrice a week previously to complete cessation of episodes when followed up for another month. Her nighttime sleep duration rose to five to six hours with only one waking episode every night. Her Epworth Sleepiness Scale score fell from 18/24 to 7/24, indicating reduced daytime sleepiness. Her tremors decreased slightly while her rigidity improved minimally.

## Discussion

We reported the case of an elderly female with PD who developed insomnia and refractory RLS besides the motor symptoms. There was a significant improvement in her insomnia, complete cessation of RLS symptoms, and a slight reduction in the motor symptoms after *Withania somnifera* was added to the existing therapy.

Sleep disorders commonly seen in PD are insomnia (including sleep latency, sleep maintenance difficulties (74-88%), early awakening), rapid eye movement (REM) behavior disorder (15-50%), sleep-disordered breathing (50%), restless legs syndrome/Willis-Ekbom disease (15%), vivid dreaming (30%), excessive daytime sleepiness (15-50%), sleep attacks (0-30%) [2].

RLS developed in our case possibly due to dopaminergic dysregulation and depletion of iron stores. Non-ergot dopamine agonists (ropinirole, pramipexole) have been shown to relieve RLS symptoms [5]. This suggests a possible link between the degeneration of dopaminergic neurons in PD and RLS. Current pharmacotherapy relies on neurotransmitter replenishment and is only effective until irreversible nerve damage sets in. Hence, our patient's symptoms became refractory with progressive neurodegeneration.

The lack of response to monotherapy (alpha-2-delta ligands like pregabalin or dopamine agonists) as in our case is termed refractory RLS. Management of refractory RLS is challenging, especially in PD, due to an inability to add dopaminergic therapy to the pre-existing levodopa [6]. This is because, over time, dopamine augmentation worsens the symptoms, increases sleep latency, and risks the development of impulse control disorders [6]. Although *Withania somnifera* was added to improve the patient’s insomnia, it may have worked as an adjunct to relieve her RLS symptoms. The wearing-off of levodopa-carbidopa effect, the limited scope of iron replenishment, reduced response to pregabalin, and the inability to add further dopaminergic agents to PD treatment could possibly make a case for the benefits of the adjunct.

**Figure 1:**
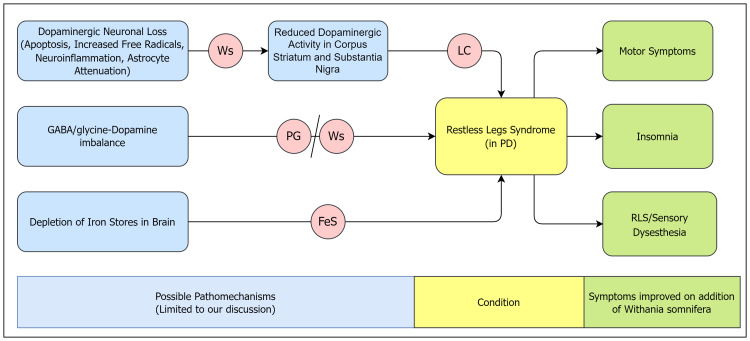
Pathomechanisms of restless legs syndrome in Parkinson's disease countered by Withania somnifera FeS: ferrous sulfate; GABA: γ-aminobutyric acid; LC: levodopa-carbidopa; PD: Parkinson's disease; PG: pregabalin; RLS: restless legs syndrome; Ws: *Withania somnifera*

*Withania somnifera* (Ashwagandha/Indian Ginseng) is a plant-derived neuroprotective agent. It is believed to improve sleep (somnifera: sleep inducer) by stress reduction. It has two major active biomolecules: withaferin-A and withanolide-A, and has previously shown pro-dopaminergic and neuroprotective actions in PD [3]. The pathomechanisms that are the possible targets of *Withania somnifera* action in our case are discussed below and illustrated separately (Figure 1).

Swiss Albino mice treated with maneb and paraquat developed oxidative damage to SNPc that was prevented by *Withania somnifera* by decreasing free radicals, increasing B-cell lymphoma-2 (BCL-2), reducing BCL-2 associated X protein (BAX), which prevented apoptosis and attenuation of activated astrocytes that protected dopaminergic neurons [3].

In experimental models, dopaminergic diencephalospinal neurons (A11-DA) have been shown to tonically inhibit spinal sensory neurons during wakefulness. During REM sleep, these inhibitory signals are in turn inhibited by γ-aminobutyric acid (GABA) and glycine-secreting neurons. When A11-DA neurons become dysfunctional in PD, the unopposed inhibition by the latter causes sensory dysesthesia, which is typical of RLS in PD [8]. Experimental evidence has shown that administration of *Withania somnifera* increases dopamine levels in the midbrain and corpus striatum [3,9]. This could counter the GABA/glycine-dopamine imbalance as explained above.

The reduction in motor symptoms after the addition of *Withania somnifera* is due to its neuroprotective effect and the resultant improvement in native dopamine activity [3]. Improvement in insomnia, however, has also been attributed to stimulation of GABA_A_ receptors by *Withania somnifera* [10].

Our case provides a nidus for research on the scope of *Withania somnifera* in idiopathic RLS in PD. Future randomized control trials must compare the efficacy of *Withania somnifera* with that of known dopamine agonists like ropinirole and pramipexole. Whether *Withania somnifera* can be added while initiating pregabalin therapy or when its efficacy declines must be studied. Evaluation of the safety and tolerance of *Withania somnifera* will be critical to its addition to the limited pharmacopeia of RLS.

## Conclusions

As PD progresses, the symptoms worsen and become refractory to medications targeting neurotransmitters. When reported, this was the first evidence where addition of Withania somnifera possibly resulted in complete remission of refractory RLS in a case of PD. Additionally, there was partial remission of insomnia and motor symptoms. Due to its neuroprotective and pro-dopaminergic properties, *Withania somnifera* could stall the symptom progression and prove to be a useful adjuvant, especially in advanced and refractory PD cases.
